# Breast cancer brain metastases: biology and new clinical perspectives

**DOI:** 10.1186/s13058-015-0665-1

**Published:** 2016-01-19

**Authors:** Isabell Witzel, Leticia Oliveira-Ferrer, Klaus Pantel, Volkmar Müller, Harriet Wikman

**Affiliations:** Department of Gynecology, University Medical Center Hamburg-Eppendorf, Martinistraße 52, 20246 Hamburg, Germany; Institute of Tumour Biology, University Medical Center Hamburg-Eppendorf, Center of Experimental Medicine, Martinistraße 52, 20246 Hamburg, Germany

## Abstract

Because of improvements in the treatment of patients with metastatic breast cancer, the development of brain metastases (BM) has become a major limitation of life expectancy and quality of life for many breast cancer patients. The improvement of management strategies for BM is thus an important clinical challenge, especially among high-risk patients such as human epidermal growth factor receptor 2-positive and triple-negative patients. However, the formation of BM as a multistep process is thus far poorly understood. To grow in the brain, single tumor cells must pass through the tight blood–brain barrier (BBB). The BBB represents an obstacle for circulating tumor cells entering the brain, but it also plays a protective role against immune cell and toxic agents once metastatic cells have colonized the cerebral compartment. Furthermore, animal studies have shown that, after passing the BBB, the tumor cells not only require close contact with endothelial cells but also interact closely with many different brain residential cells. Thus, in addition to a genetic predisposition of the tumor cells, cellular adaptation processes within the new microenvironment may also determine the ability of a tumor cell to metastasize. In this review, we summarize the biology of breast cancer that has spread into the brain and discuss the implications for current and potential future treatment strategies.

## Background

Because of improvements in the treatment of patients with metastatic breast cancer, long-term survival can be achieved. Nevertheless, 15–30 % of patients with metastatic breast cancer will develop brain metastases (BM) during the course of the disease [1]. BM are not only associated with an extremely poor prognosis but also with neurological impairments by often affecting both cognitive and sensory functions [2]. Therefore, BM have become a major limitation of life expectancy and quality of life in many patients. The development of management strategies for BM is thus an important clinical challenge.

**Fig. 1:**
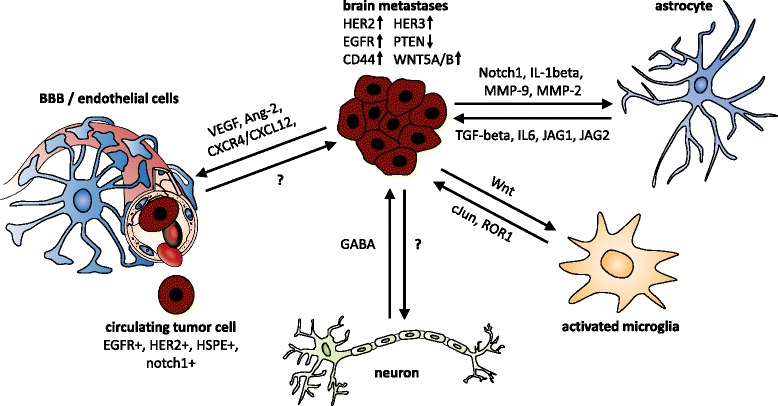
Schematic of tumor cell interactions in the brain. An intensive direct and indirect cross-talk between the resident cells and tumor cells needs to occur for circulating tumor cells to pass through the BBB and grow in the brain. These interactions, in addition to the genetic predisposition of the tumor cells, result in a multitude of pathway activations in both tumor and host cells. *BBB* blood–brain barrier, *EGFR* epidermal growth factor receptor, *GABA* gamma-aminobutyric acid, *HER* human epidermal growth factor receptor, *IL* interleukin, *JAG* jagged, *MMP* matrix metalloproteinase, *TGF* transforming growth factor, *VEGF* vascular endothelial growth factor

Breast cancer is the second most common cause for the development of BM after lung cancer. Lung and breast cancer BM are more commonly diagnosed than primary brain tumors. The incidence of BM in breast cancer patients is rising, probably because many patients survive longer due to the improvement of systemic therapies to control extracranial disease; thus, patients can experience BM before dying from other manifestations. This reflects an insufficient control of cerebral tumor spread by current treatment strategies. Moreover, detection rates of subclinical BM increase with improved imaging techniques via contrast-enhanced magnetic resonance imaging (MRI) as a standard of care in diagnosing BM (Table 1).

**Table 1:** Frequency of site-specific metastasis among metastatic breast cancer patients

Site of relapse	Brain (%)	Bone (%)	Lung (%)	Liver (%)	Pleura (%)
Autopsy cases^a^					
Median	21	71	71	62	50
Range	15–35	50–74	60–80	50–71	35–80
All subtypes^b^	12–17	48–62	23–32	15–27	7–31
Luminal A	8–15	65–67	6–7	12–29	15–28
Luminal B	11	58–71	24–30	4–32	11–35
TNBC/basal	25–27	17–39	40–43	13–21	3–29
HER2-positive	11–20	61–62	15–42	22–44	0–32

^a^Median value and range from seven different studies reported by [85, 86]

^b^Summarized data from the studies reported in [11, 12, 14]

*HER* human epidermal growth factor receptor, *TNBC* triple-negative breast cancer

Distant metastasis formation is a multistep process and is often referred to as the metastatic cascade. Animal studies have shown that only a very small percentage of tumor cells are capable of completing the various steps; the most limiting of which is the outgrowth of tumor cells at distant sites [3]. The ability of tumor cells to initiate growth (e.g., in the brain) is probably largely dependent on cross-talk between tumor and brain resident cells. Additionally, a genetic predisposition of cellular adaptation processes within the new microenvironment may play an important role. Understanding the biology of BM is important for both the prediction of patients at risk to develop BM and the discovery of new drug targets.

## Epidemiology, incidence, and risk factors

Several factors for an increased risk of BM have been identified in a breast cancer scenario. Younger patients, poorly differentiated tumors (high grade), hormone receptor-negative status, and four or more metastatic lymph nodes have been associated with increased BM risk [1]. Human epidermal growth factor receptor (HER)2-positive and triple-negative breast cancer (TNBC) patients also have a higher risk of BM compared with luminal cancer patients [4, 5]. In HER2-positive and TNBC patients, incidences of BM as high as 30–40 % have been described (Table 1) [4–6].

Survival rates after cerebral metastasis differ depending on prognostic factors, tumor subtype, Karnofsky performance status, and treatment [2]. Despite the use of neurosurgery and radiotherapy, few patients live longer than 1 year [2, 7]. As in a primary tumor setting, patients with a triple-negative tumor have the worst prognosis. In a retrospective study by Niikura et al. [7] with 1256 patients diagnosed with BM, the median overall survival (OS) was 8.7 months (95 % confidence interval (CI): 7.8–9.6). However, when the cohort was stratified according to tumor subtype, patients with luminal tumors had an OS of 9.3 months (95 % CI: 7.2–11.3) and those with HER2-positive tumors had an OS of 16.5 months (95 % CI: 9.1–13.8); the OS for patients with triple-negative tumors was only 4.9 months (95 % CI: 3.9–5.9).

## Site-specific metastasis and breast cancer subtypes

Steven Paget [8] proposed the so-called “seed and soil” theory more than 120 years ago and described that the nonrandom spread of tumors is dependent on the interactions between metastatic tumor cells (“seed”) and their organ microenvironment (“soil”). Numerous studies since then have supported and confirmed this hypothesis. In the 1970s, Fidler and Kripke [9] showed in experimental metastasis assays that even though tumor cells can reach the vasculature of all organs, metastases developed only in specific organs, demonstrating that the outcome of metastasis is dependent on the cross-talk between tumor cells and host tissue.

Regarding BM, lung, breast, and melanoma tumor cells appear to have a propensity towards the brain, whereas prostate cancer rarely metastasizes to the brain [10]. Interestingly, although lung adenocarcinomas and breast cancer share similar organ tropism (metastatic patterns), they have strikingly different metastatic rates [1]. In lung cancer BM occur usually within 2 years after the primary diagnosis, whereas in breast cancer BM are usually associated with the metastatic stage of disease and may occur a decade after primary diagnosis and successful treatment. However, in TNBC patients BM are often detected rather early in the course of the disease.

Different breast cancer subtypes have a very different likelihood of metastasizing to the brain [11–13] (Tables 1 and 2). Kennecke et al. [12] investigated the metastatic patterns in 3726 early-stage breast cancer patients diagnosed between 1986 and 1992. The luminal tumors were significantly associated with a bone-seeking phenotype and were less frequently observed in patients with lung and brain metastases. Patients with HER2-positive and basal-like tumors had a 5.3-fold to 3.6-fold increased risk to develop BM compared with those with luminal A tumors. In a similar study by Smid et al. [14], 344 primary breast tumors were investigated from lymph node-negative patients without adjuvant treatment. Again, luminal tumors were characterized by bone metastases, whereas BM were most commonly found among HER2 and basal-like breast cancer. The gene expression profiling of these primary tumors showed that both basal-like tumors as well as brain metastatic tissue (irrespective of breast cancer subtype) showed upregulation of WNT signaling and upregulation of the genes involved in cell cycle control [14]. These results showed that although subtypes differ in their molecular characteristics, they share certain biological features when they have similar metastatic patterns.

**Table 2:** Reported frequencies for the first site of metastasis among breast cancer patients

Site of metastasis	Brain (%)	Bone (%)	Lung (%)	Liver (%)	Pleura (%)
All^a^	7–16	40–51	13–22	6–18	ND
Luminal A	2	47	8	18	7
Luminal B	0	35	16	12	12
TNBC	10	29	21	10	7
HER2-positive	2	29	23	27	8

^a^Summarized data from the studies reported in [13, 87, 88]

*HER* human epidermal growth factor receptor, *ND* not determined, *TNBC* triple-negative breast cancer

A large study by Sihto et al. [13] investigated the associations between the protein expression levels of 18 different breast cancer-related proteins in the primary tumor and the first site of metastasis. In this study, tumors from patients with the brain as the first metastatic site were negative for estrogen and progesterone receptor but frequently expressed CK5, nestin, and prominin-1 (CD133). Interestingly, both nestin and CD133 are considered to be cancer stem cell (CSC) markers for glioblastoma [15]. Similarly, an in vitro selection of a CSC population from the TNBC cell line GI-101 identified CD133 and CD44 as marker proteins for these cells. These cells were shown to be fivefold more invasive than the parental cells, and mice injected with the cells had significantly more BM and shorter OS [16].

Taken together, common biological features among triple-negative and HER2-positive tumors support the survival and growth of these tumors in the brain microenvironment compared with hormone receptor-positive tumors. These features could be exploited as targets for future therapeutic strategies.

## Local and systemic treatment of BM

Historically, breast cancer BM were treated with whole-brain radiation therapy (WBRT) or with surgery if possible. The role of WBRT after the surgical resection of a single metastasis has been well established for controlling local recurrence [17]. However, the use of WBRT has come under recent criticism because of the possibility of neurocognitive decline related to brain radiation [18].

The rise of stereotactic radiosurgery has become an alternative for patients with limited disease due to its advantage of a single-session delivery and a minimal delay for systemic therapy. Both local therapy and systemic treatment enhance OS [19]. In 420 patients with BM receiving WBRT, the median survival in a scenario of BM in patients without and with systemic treatment after WBRT was 3 and 10 months, respectively (*P* < 0.0001). No survival benefit for systemic treatment was observed only in the triple-negative subset. In all other subgroups (HER2-positive, luminal A and B), a survival benefit from systemic treatment could be achieved.

Most cytotoxic agents do not cross the blood–brain barrier (BBB), and the presence of BM has been an exclusion criterion for nearly all clinical trials on treatment in metastatic breast cancer. Studies examining chemotherapy regimens usually included various solid tumors and reported response rates of between 4 and 38 % [20]. Therefore, it is largely unclear how existing therapies function in patients with BM; there is thus no global consensus regarding the ideal treatment strategy.

Although trastuzumab is effective for treating HER2-positive breast cancer, it is not clear whether it also acts in the brain. A study by Stemmler et al. examined trastuzumab levels in serum and cerebrospinal fluid. Prior to radiotherapy, the median serum versus cerebrospinal trastuzumab level ratio was 420:1. After the completion of radiotherapy, the ratio was 76:1. In patients with concomitant meningeal carcinomatosis, this ratio was 49:1 [21].

In more recent publications, a higher incidence of BM was reported in HER2-positive patients treated with trastuzumab [22]. Data analysis showed that the enhanced risk of BM after trastuzumab treatment was due to an improved systemic control of the disease. The continuation of trastuzumab treatment in patients with BM is beneficial; however, it is unclear whether this benefit is due to drug efficacy in the brain or better systemic control [5].

The small molecule kinase inhibitor of epidermal growth factor receptor (EGFR) and HER2, lapatinib, was presumed to be able to cross the BBB. In findings from a mouse model, the average lapatinib concentration in BM was only 10–20 % of that in peripheral metastases. Only in a subset of brain lesions (17 %) did the lapatinib concentration approach that of systemic metastases [23]. In HER2-positive breast cancer patients who progressed after WBRT, lapatinib monotherapy showed minor activity as a single agent [24]. In 39 patients, only one partial response was observed. In another study, lapatinib monotherapy showed a response of 6 % [25]. Interestingly, the addition of capecitabine increased the response rates to 20 %. In the LANDSCAPE trial [25], the combination of lapatinib and capecitabine administered prior to WBRT in newly diagnosed HER2-positive BM revealed a central nervous system (CNS) response rate of 67 %. A study in patients with resected BM found that capecitabine and lapatinib penetrate to a significant although variable degree into the brain and that drug delivery to BM is variable and in many cases appears partially limiting [26]. A recent report from a study found that the incidence of BM as the first site of relapse was 3 % for the combination of lapatinib–capecitabine and 5 % for trastuzumab–capecitabine [27]. These data do not support a better activity for lapatinib compared with trastuzumab in the prevention of BM.

Despite these reports suggesting the direct activity of agents in the brain, chemotherapy is generally prescribed secondary to surgery or radiotherapy.

Recent clinical findings showed some efficacy of antibody-based therapy in BM with trastuzumab-DM1 (T-DM1) [28, 29]. In the EMILIA trial [30], T-DM1 was associated with significantly improved OS compared with lapatinib and capecitabine in patients with asymptomatic BM at baseline.

The efficacy of bevacizumab (a vascular endothelial growth factor (VEGF) antibody) in combination with radiotherapy and chemotherapy was investigated in 35 patients with a CNS-objective response in 13 patients (37.1 %) [31]. Additionally, the combination of WBRT and bevacizumab without chemotherapy showed some efficacy in a phase I trial including 13 patients with breast cancer BM [32].

Some agents show moderate activity in the brain; however, tumor cells that manage to spread into the brain have other resistance mechanisms. Therefore, it would be of high interest to understand the nature of breast cancer tumor cells that outgrow in the brain in an effort to prevent this process.

## Signaling pathways involved in breast cancer brain metastasis

Among the different pathways associated with breast cancer BM formation, we will briefly discuss two stem cell pathways (Wnt and Notch) as well as the EGFR pathway (ERBB) because substantial evidence for the involvement of these pathways in BM has been obtained (Fig. 1).

CSCs have been extensively discussed in the literature as the initiators of tumor growth and metastasis. Both Notch signaling and Wnt signaling are evolutionarily conserved and important for normal stem cell function but are also often associated with CSCs and found to be deregulated in cancers such as glioblastomas [33]. Nam et al. investigated the expression profiles of different metastatic variants of the MDA-MB-435 breast cancer cell line. The cell line with enhanced BM properties had activated Notch pathway via notch1 and jagged-2 (JAG2) [34]. Xing et al. showed that breast tumor cells in the brain highly express interleukin (IL)-1β, which in turn can activate the surrounding astrocytes to express jagged-1 (JAG1). This direct interaction of the activated astrocytes and CSCs significantly stimulated Notch signaling in CSCs [35]. Similarly, McGowan et al. showed that when notch1 was silenced in the MDA-MB-231 brain-seeking cells, the CD44^hi^/CD24^low^ phenotype was reduced, which in turn led to fewer macrometastases in the brain when injected into nude mice [36].

Several studies have investigated the clinical and functional roles of EGFRs (EGFR, HER2, HER3) and their downstream mediators (PTEN, PIK3CA, AKT, mTOR) in breast cancer BM [37–40]. We performed a genome-wide screening of the genomic and transcriptional aberration patterns in BM from breast cancer patients and showed that BM, in general, display similar chromosomal aberrations to those of primary tumors but with a notably higher frequency [41]. Seven specific regions were more commonly aborted in the BM. Protein kinase C, delta binding protein (*PRKCDBP*) was identified as the potential target gene in 11p15 loss, whereas EGFR was the driver for a narrow gain of 7p12 and PTEN a driver for the loss of 10q23 [41]. Further studies identified two separate pathways (EGFR/PTEN) among triple-negative patients and HER2-positive patients, both leading to a significantly increased risk of BM [42]. Primary tumors from patients with a brain relapse showed similar high aberration frequencies compared with those of BM samples, whereas these aberrations were rarely found among patients with bone metastases. Interestingly, mutations in both *PTEN* and *EGFR* are described as the main driver alterations for the very aggressive glioblastoma; therefore, the data imply that these aberrations (according to the seed and soil theory) provide the necessary growth and survival signaling for tumor cells in the brain environment. Functional studies have shown that EGFR plays an important role in breast tumor cell migration and invasion to the brain, whereas proliferation is less influenced [43].

As previously discussed, data have clearly shown that HER2 overexpression is associated with BM [5]. In contrast to EGFR, mouse experiments have indicated that HER2 overexpression may especially increase the outgrowth of BM [44]. Additionally, HER3 overexpression has been associated with BM in breast cancer patients [45]. In contrast to EGFR and HER2, HER3 expression seems to be often induced in the BM [46]. Heregulin is the primary ligand of HER3/HER2 heterodimers and is highly expressed in the human brain. Heregulin has been shown to induce the transendothelial migration of HER2/HER3-positive breast cancer cell lines across a tight barrier of primary brain microvascular endothelia. Again, MMP-9 was indicated as one of the factors partially mediating this process [47]. Interestingly, heregulin treatment suppressed the expression of *RECK*, a gene found to be downregulated in breast cancer BM [48].

Breast cancer patients with BM show less circulating tumor cells (CTCs) when detected via epithelial-like properties compared with breast cancer patients with other metastases [49]. Zhang et al. [50] identified that the CTCs which expressed HER2, EGFR, HSPE, and notch1 but not EpCAM were the most aggressive and capable of generating BM in nude mice, again indicating that both the ERBB and notch pathways may be crucial for BM. There is intensive bidirectional crosstalk between the ERBB and notch pathways in breast cancer, indicating that the simultaneous inhibition of both ERBB and notch pathways may be an interesting option in treating breast cancer BM patients.

## Brain microenvironment and breast cancer

### BBB and blood–tumor barrier

The controversial concept of the BBB has been discussed and has been continuously developed since the first experimental evidence in 1885. Currently, the BBB is defined as a selective diffusion barrier at the level of the cerebral microvascular endothelium that is characterized by the lack of fenestrations and the presence of tight junctions (TJs) on endothelial cells [51].

Under normal physiological conditions, this cellular barrier selectively regulates the exchange between blood and brain compartments by preventing the paracellular diffusion of hydrophilic compounds, thereby mediating the transport of nutrients to the brain, effluxing potentially toxic substances from the cerebral compartment, and excluding the transendothelial migration of blood cells. The latter primarily represents an obstacle that stops CTCs from entering the CNS; however, the restrictive features of the BBB play a protective role against immune cell and toxic agents once metastatic cells have colonized the cerebral compartment. Recent studies have indicated that, in addition to providing a cellular barrier, brain endothelial cells can actively support tumor cell growth and invasion [52]. The BBB was also found to be responsible for the fact that patients with primary brain tumors rarely have extracranial metastases and present low levels of circulating tumor DNA [53]. However, others and we recently detected CTCs in the blood of approximately 20 % of glioma patients at primary diagnosis [54, 55]. Thus, it is unclear to what extent the BBB actually shields the brain from the periphery in cancer patients. Notably, glioma data may not be equated directly with brain metastasis data because of varying biologies. Moreover, whether a tumor cell can pass through the BBB can be vastly different from whether a drug can penetrate the BBB at a sustained, high level.

#### Tumor cell extravasation

Tumor cell extravasation in the brain occurs preferentially through paracellular transmigration (through endothelial junctions) rather than transcellular transmigration (through single endothelial cells) [56]. Endothelial cell junctions are the part of the BBB most likely to be modified in pathological situations, including BM formation. Several factors have been described to play a key role in this process. VEGF may contribute to BM formation by enhancing the transendothelial migration of tumor cells through the downregulation of endothelial integrity [57]. Additionally, CD44 has been described as a key mediator of the transendothelial migration of breast cancer cells. Here, glycosaminoglycan hyaluronan binds to its receptor CD44 at the surface of the neoplastic cells and subsequently cross-links with activated CD44 receptors expressed on the endothelium, suggesting that cancer cells with elevated hyaluronic acid synthase activity and high CD44 expression exhibit an increased potential to metastasize [58, 59]. Angiopoietin-2 has recently been shown to mediate BBB impairment and the colonization of TNBC cells in the brain by increasing brain vascular permeability and changes in Zonula occludens ZO-1 and claudin-5 TJ protein structures [60]. Chemokine CXCL12 expressed in the brain and its counter-receptor CXCR4, which is present on the surface of breast tumor cells, have been suggested to play essential roles in tumor cell migration in the brain that could be prevented by blocking the CXCR4-dependent intracellular pathway [61]. Gene expression analyses of cells with high BM activity and subsequent functional analyses identified the cyclooxygenase COX2, the EGFR ligand HBEGF, and the α2,6-sialyltransferase ST6GALNAC5 as mediators of cancer cell passage through the BBB [62].

#### Blood–tumor barrier

The BBB is frequently disrupted after BM formation and is defined as the blood–tumor barrier (BTB). However, MRI data have shown that not all BM display elevated BTB permeability [63, 64]. BM of TNBC or basal-type breast cancers may disrupt the BBB, whereas the BM of HER2-positive breast cancers tend to preserve it [65]. There are few data on the impact of BBB breakdown and efficient drug delivery in a scenario of breast cancer BM. In a preclinical study using two different models of breast cancer BM, most metastases exhibited some increased BTB permeability; however, BTB permeability remained poorly correlated with lesion size, and only ∼ 10 % of lesions with the highest permeability exhibited cytotoxic responses to paclitaxel or doxorubicin [66].

Unfortunately, few approaches have been attempted to overcome poor drug distribution to metastatic lesions.

### Brain colonization, angiogenesis, and vessel cooption

In recent years, a variety of experimental studies have demonstrated that disseminated tumor cells in the brain further interact with the capillary walls after extravasation by attaching to the abluminal side of existing vessels and growing along them [52, 67, 68]. This process, known as vascular cooption, implies that such tumor cells do not require phenotypic changes (i.e., angiogenic switch) during brain colonization. Here, the cell adhesion molecule L1CAM mediates the metastatic spread of breast cancer cells on the vasculature as well as interactions between cancer cells [69]. Once tumor cells have infiltrated the brain, they require an adequate blood supply to grow and develop a metastatic lesion. The mechanisms that are involved in blood vessel recruitment by BM cells appear to be strongly dependent on tumor origin as well as the metastatic microenvironment [52, 70]. Preclinical models have shown that BM can grow to a certain size only through coopting preexisting vessels; later, the blood supply of macrometastases can be obtained through either angiogenesis activation (lung carcinoma model) or persistent vessel cooption (melanoma model) [52].

In a mouse breast cancer BM model, the authors observed two different phenotypes of metastatic lesions growing either within the parenchyma or in the leptomeninges. Both of these phenotypes showed characteristic blood vessel architectures. In intraparenchymal lesions, tumor cell growth was primarily observed around small blood vessels (cooption), whereas larger vessels surrounded by smooth muscle cells were only identified in dense solid leptomeningeal lesions (angiogenesis) [68]. Indeed, the “soil” for malignant cells in the CNS has been suggested to be vascular rather than neuronal [67]. Here, the vascular-induced adhesion and invasion of breast cancer cells may be sufficient for tumor growth prior to angiogenesis, and a β1-integrin subunit has been described to play a key role during these processes.

In contrast, a different preclinical study has indicated a key role for angiogenesis in the formation and development of BM in breast cancer. The authors of that study showed a significant increase in VEGF-A production in a BM breast cancer cell line (MDA-MB-231-BR) compared with the parental cell line (MDA-MB-231) that corresponds to BM lesions with significantly more CD31-positive blood vessels following intracarotid injection in mice [71]. In a recent preclinical mouse tumor model mimicking postsurgical adjuvant or metastatic therapy, a VEGF pathway-targeting antibody drug (bevacizumab) with chemotherapy (paclitaxel) resulted in antitumor activity in a metastatic setting [72]. Based on these data and those already mentioned, preliminary clinical studies are ongoing and have shown some efficacy in the therapeutic role of targeting VEGF in combination with chemotherapy or radiation therapy [32, 73, 74].

### Tumor cell interactions with residential brain cells

The brain is a unique organ that is well shielded from the rest of the body by the BBB. The most abundant cells in the brain are glial cells and neurons. Several studies have recently shown the important role of different glial cells in metastatic processes; however, the role of neurons has not thus far been investigated in detail [75].

Neurons transmit information through neurotransmitters. Gamma-aminobutyric acid (GABA) is one the neurotransmitters at synaptic junctions. Neman et al. [76] recently showed an upregulation of GABA transporters and GABA receptor in BM that led to an increased uptake of the neurotransmitter, increased NADPH production, and a proliferation advantage conferred to breast tumor cells.

There are various glial cell types in the brain, among which two have been associated with BM (i.e., astrocytes and microglia). Astrocytes are nonproliferative in the normal adult brain; however, upon injury these cells can be activated, which can lead to glial scar formation or gliosis [75]. BM also induce the strong local activation of astrocytes including reactive gliosis [75]. In breast cancer patients, the activated astrocytes accumulate both around and inside the metastatic foci [24] with the tumor cells forming direct contact with the astrocytes [77]. The reactive astrocytes secrete a multitude of chemokines, cytokines, and ILs. Many of these factors, such as IL-6 and transforming growth factor beta, can function as oncogenic signals for the tumor cells [75, 78]. Coculture experiments with astrocytes have shown that factors secreted by the astrocytes induce both the migration and invasion of breast cancer cells [79]. Wang et al. [79] identified two matrix metalloproteases (MMP-2 and MMP-9) to be one of the mediators for astrocyte-induced tumor cell invasion. Furthermore, when breast cancer cells are cocultured with astrocytes, the tumor cells become more resistant to cisplatin treatment. The protective function of astrocytes is dependent on direct contact between the cells; indirect cocultures do not provide protection against tumor cell death [80].

Microglia are brain-specific macrophages involved in brain defense. The role of microglia in BM is less well understood despite the fact that inflammation is defined as one of the hallmarks of cancer, and the role of differentially activated macrophages in cancer progression is widely investigated. In a manner similar to astrocytes and macrophages, microglia are usually in a quiescent state and when activated can perform diverse functions [81]. Similar to astrocytes, microglia have been shown to enhance the invasion and colonization of brain tissue by breast cancer cells. This invasion was shown to be dependent on the activation of Wnt signaling, and the Wnt inhibitor Dickkopf-2 nearly completely abolished the microglia-induced invasion of tumor cells [82].

These studies clearly show that an intensive direct and indirect cross-talk between the resident cells and tumor cells occurs when cells arrive in the brain, resulting in a multitude of different pathway activations in both tumor and host cells. Furthermore, the tumor cells that are able to grow in the brain appear to have gained the ability to exploit the brain endogenous substrates that are secreted by the resident cells as oncogenic signals.

## Conclusion

In recent decades, a substantial improvement in the treatment of breast cancer patients, including those in the metastatic situation, has been achieved. In this context, patients with BM represent an exception. Despite the increasing and important clinical BM problem, knowledge about the mechanisms of cerebral metastasis development and optimal treatment strategies remains limited and substantially less frequently investigated than, for example, bone metastasis formation. Almost no prospective trial data are available for the efficacy of systemic therapy in patients with BM; thus, the optimal strategy for these patients is unclear. To obtain further knowledge about outcomes in breast cancer patients, we established a national clinical data registry and tumor bank in Germany [83]. Similar efforts are in the making in other countries.

New markers for predicting BM occurrence in the primary tumor setting are urgently needed for the early detection of high-risk patients and to effectively prevent the formation of BM in those patients. To what extent “liquid biopsies” (i.e., analysis of CTCs or circulating nucleic acids) may contribute to this goal remains under investigation [84]. In principle, it would be highly desirable to track changes in BM during therapy via sequential blood analyses.

Understanding the molecular changes that breast tumor cells undergo to successfully colonize the brain compartment is crucial in obtaining novel and BM-specific therapeutics. In this context, targeting interactions between disseminated breast tumor cells and residential brain cells may represent a promising approach.

Finally, effective drug delivery into the brain still represents a key challenge. Here, novel therapeutic strategies for the treatment of brain tumors, such as the modulation/destruction of BBB components and transporter systems or nanotherapy, may also be appropriate for breast cancer BM.

## Abbreviations

BBB: blood–brain barrier
BM: brain metastases
BTB: blood–tumor barrier
CI: confidence interval
CNS: central nervous system
CSC: cancer stem cell
CTC: circulating tumor cell
EGFR: epidermal growth factor receptor
GABA: gamma-aminobutyric acid
HER: human epidermal growth factor receptor
IL: interleukin
MRI: magnetic resonance imaging
OS: overall survival
T-DM1: trastuzumab-DM1
TJ: tight junction
TNBC: triple-negative breast cancer
VEGF: vascular endothelial growth factor
WBRT: whole-brain radiation therapy
